# Folic Acid Alleviates X‐Ray Irradiation‐Induced Jaw Malformation in Zebrafish

**DOI:** 10.1002/cga.70069

**Published:** 2026-07-01

**Authors:** Tanisha Das, Takashi Shiromizu, Aina Higuchi, Sakyo Yasojima, Misaki Morimoto, Miho Hosaka, Makoto Kashima, Junko Koiwa, Yuhei Nishimura

**Affiliations:** ^1^ Department of Integrative Pharmacology Mie University Graduate School of Medicine Tsu Mie Japan; ^2^ Department of Chemistry and Biological Science College of Science and Engineering, Aoyama Gakuin University Sagamihara Kanagawa Japan; ^3^ Department of Molecular Biology Faculty of Science, Toho University Chiba Japan

**Keywords:** folic acid, gene expression, jaw malformation, oxidative stress, X‐ray irradiation

## Abstract

X‐rays are a form of ionizing radiation that has sufficient energy to remove electrons from atoms, thereby creating potentially harmful ions. X‐ray irradiation during organogenesis can have profound detrimental effects, depending on the developmental stage and irradiating energy. In this study, we examined the impact of X‐ray irradiation on zebrafish development at 36 h post‐fertilization and observed marked jaw malformation, which was alleviated by folic acid pretreatment. Mechanistic studies revealed that folic acid pretreatment suppressed irradiation‐induced production of reactive oxygen species. Transcriptome analysis performed at 24 and 48 h post‐irradiation revealed dysregulated expression of apolipoprotein A‐IV a (*apoa4a*), methionine adenosyltransferase 1A (*mat1a*), heat shock protein 90 alpha class A member 1 tandem duplicate 1 (*hsp90aa1.1*), FKBP prolyl isomerase 5 (*fkbp5*), plac8 onzin related protein 3 (*ponzr3*), and prostaglandin E synthase 3a (*ptges3a*), which was not observed in zebrafish treated with folic acid before the irradiation. These findings suggest that folic acid alleviates X‐ray irradiation‐induced jaw malformation in zebrafish, at least in part, by reducing oxidative stress and ameliorating dysregulated gene expression.

## 1 Introduction

Craniofacial anomalies are defects associated with the development and differentiation of soft tissues and bony structures of the head and neck, and they usually arise from disturbances at one or more stages of early embryogenesis. The most common craniofacial defects in humans are cleft palate, cleft lip, and orofacial clefts, with cleft lip and/or palate estimated to occur in approximately 1 in 700 live births [1, 2]. Other common defects include hemifacial microsomia, craniosynostosis, and holoprosencephaly [3]. The defects are typically associated with dysregulation of neural crest cell development, particularly impairment in the formation, migration, or differentiation of the cells [4, 5]. Multiple causes of craniofacial anomalies have been identified, including genetic mutations, alcohol, folic acid (FA) deficiency, infections, gestational diabetes, and pharmaceutical agents, as well as environmental teratogens such as ionizing radiation [6, 7].

X‐rays (XR), the most common forms of ionizing radiation, have extensive medical applications, including diagnostic techniques and radiotherapies [8]. However, exposure to XR during the early stages of embryogenesis can result in developmental defects, largely due to the generation of reactive oxygen species (ROS), oxidative stress, and DNA damage in rapidly differentiating cells [9]. Given that the critical period of organogenesis in humans occurs at 3–8 weeks of gestation, when many women may be unaware of the pregnancy, it is essential that efforts are made to identify strategies that protect against XR‐induced developmental abnormalities.

FA is an essential water‐soluble B vitamin and a widely recommended supplement for pregnant women to prevent neural tube defects in the fetus [10]. FA plays an essential role in one‐carbon metabolism, a process through which single‐carbon units such as methyl and formyl groups are transferred during critical processes such as DNA synthesis and repair [11]. Additionally, FA has antioxidant properties, acting as a protectant against harmful free radicals and reducing the oxidative damage that results from exposure to ionizing radiation [12]. However, whether or not FA supplementation can protect against radiation‐induced developmental injury, specifically craniofacial development, has not yet been fully elucidated [13].

To address this, we examined the effects of XR irradiation on jaw development in zebrafish embryos and investigated the impact of pretreatment with FA on XR‐induced defects. Zebrafish have many attributes that make them an excellent model for studies of vertebrate development and have been widely utilized for determining the etiology of craniofacial anomalies [14]. Not only does the optical transparency of zebrafish allow precise spatiotemporal monitoring of key events such as protein and gene expression, but zebrafish also share many of the molecular pathways that regulate craniofacial morphogenesis with higher vertebrates, making them a relevant model for evaluating the effects of environmental factors and therapeutic and/or protective interventions on these processes [15]. Thus, we also evaluated the potential mechanisms underlying XR‐induced effects in zebrafish.

## 2 Materials and Methods

### 2.1 Ethics Statement

This study was approved by the Institutional Animal Care and Use Committee at Mie University (no. 2020–19). All animal experiments conformed to the ethical guidelines established by the committee.

### 2.2 Zebrafish Husbandry

An albino zebrafish line, slc45a2^b4^/^b4^, which lacks melanin pigmentation in melanophores, was obtained from the Max Planck Institute for Developmental Biology (Tübingen, Germany) and maintained as described previously [16]. Embryos were obtained via natural mating and maintained in 0.3× Danieau's solution (19.3 mM NaCl, 0.23 mM KCl, 0.13 mM MgSO_4_, 0.2 mM Ca(NO_3_)_2_, 1.7 mM HEPES, pH 7.2). Zebrafish were raised at 28.5°C ± 0.5°C and maintained under a 14 h/10 h light/dark cycle.

### 2.3 Chemicals

FA (Nacalai Tesuque, Kyoto, Japan) was dissolved in 0.3% Danieau's solution to prepare a 40 mM stock solution, and the pH was adjusted to 7.0 using 1 N sodium hydroxide (NaOH). The stock solution was then diluted 1:100 in 0.3% Danieau's solution to obtain working concentrations (200 or 400 μM) for experiments. 2′,7′‐Dichlorodihydrofluorescein diacetate (DCF‐DA; Invitrogen, Waltham, MA, USA) was dissolved in dimethyl sulfoxide to prepare a 10 mM stock solution, and the stock was then diluted in 0.3% Danieau's solution to obtain the working concentration (25 μg/mL) for experiments.

### 2.4 FA Treatment and XR Irradiation

Zebrafish embryos were randomly assigned into three groups for imaging analysis (Control, XR, and FA + XR) and four groups for transcriptome analysis (Control, XR, FA, and FA + XR). Each group was incubated in a separate well of a 6‐well plate. At 35 or 35.5 h post‐fertilization (hpf), a freshly prepared sterile solution of FA was added to the FA and FA + XR embryo groups to give a final concentration of 200 μM (for transcriptome analysis) or 400 μM (for imaging analysis), and the Control and XR groups received an equivalent volume of 0.3× Danieau's solution. After 0.5 or 1 h of preincubation, the 6‐well plates for the XR and FA + XR groups were placed in the X‐ray irradiation apparatus (Hitachi Try System Model MBR‐1618R‐BE, Hitachi Power Solutions, Hitachi, Japan) and exposed to XR (8Gy). The X‐ray irradiation was performed at a tube voltage of 150 kV and a tube current of 20 mA. It took about 5 min to irradiate 8 Gy using this setting. The embryos in all groups were then maintained in 0.3% Danieau's solution with or without FA, as appropriate, until imaging analysis at 37 and 120 hpf (1 and 84 h post‐irradiation, respectively) or transcriptome analysis at 37, 60, and 84 hpf (1, 24, and 48 h post‐irradiation, respectively).

### 2.5 Alcian Blue Staining

Alcian blue staining was performed to visualize cartilage as described previously [17]. Briefly, at 120 hpf, Control, XR, and FA + XR groups of embryos were fixed overnight at 4°C in a glass vial containing 4% phosphate‐buffered formaldehyde (4% PFA) with shaking. The next day, embryos were washed several times with phosphate‐buffered saline supplemented with 0.1% Tween‐20 (PBT) and bleached with 30% hydrogen peroxide for 2 h. The embryos were then washed twice with PBT and stained overnight with Alcian blue solution (1% concentrated hydrochloric acid [HCl], 70% ethanol [EtOH], 0.1% wt/v Alcian blue). The embryos were rinsed three times with acidic ethanol (HCl‐EtOH: 5% concentrated HCl, 70% EtOH) and rehydrated by sequential incubation in 75%:25% HCl‐EtOH: distilled H_2_O (dH_2_O); 50%:50% HCl‐EtOH: dH_2_O; 25%:75% HCl‐EtOH: dH_2_O; and 100% dH_2_O. The zebrafish were then mounted in 3% methylcellulose and imaged under a stereomicroscope (SMZ800, Nikon, Japan). Craniofacial morphometry was performed using ImageJ software (NIH, Bethesda, MS, USA), and Meckel's cartilage length and the ceratohyal angle were quantified. The experiments were done twice. Five and four larvae per group were analyzed in the first and second experiments, respectively.

### 2.6 DCF‐DA Staining

DCF‐DA staining was performed to measure ROS levels in zebrafish as described previously [18], with some modifications. At 35.5 hpf (30 min before XR irradiation), zebrafish embryos were treated with DCF‐DA (25 μg/mL) with or without FA (400 μM) in 0.3% Danieau's solution for 30 min in the dark. At 36 hpf, the zebrafish were exposed to XR (8 Gy), and 1 h later (37 hpf), the animals were imaged using an Olympus SZX10 fluorescence stereomicroscope equipped with SZX2‐ILLTQ fluorescence illumination base and MGFP filter (excitation ~470 nm, emission ~525 nm). All images were captured under the same conditions. Fluorescence intensity was quantified using ImageJ software, and fluorescence in the head region was quantified. The experiments were done three times. Eight larvae per group were analyzed in each experiment.

### 2.7 Transcriptome Analysis

At 1, 24, or 48 h post‐irradiation, total RNA was extracted from each larva using Direct‐TRI [19], and RNA concentrations were determined using a QuantiFluor RNA System Kit with a Quantus Fluorometer (Promega, Madison, WI, USA) according to the manufacturer's instructions. Six larvae were used for each group at each time point. RNA was diluted to a final concentration of 1 ng/μl and a library was prepared for RNA‐Seq as described previously [20, 21]. Sequencing of 150‐bp paired‐end reads was conducted using a HiSeq X Ten system (Illumina). Quantification of gene expression was conducted using Danio_rerio.GRCz11.cdna.all.fa as described previously [22].

Genes differentially expressed between the control, FA, XR, and FA + XR groups were analyzed using the Tag Count Comparison (TCC) package [23] in R/Bioconductor using a false discovery rate threshold of 10%. Genes with an average count of ≥ 2.5 were subjected to TCC analysis to identify differentially expressed genes. g.Profiler [24] was used for gene ontology analysis.

The transcriptome data from this study have been submitted to GEO under accession number GSE334519.

### 2.8 Statistical Analysis

Data derived from Alcian blue and DCF‐DA staining were analyzed using Prism 10 (GraphPad, La Jolla, CA, USA). Differences between the Contol, FA, and/or FA + XR groups were assessed using the Kruskal–Wallis test followed by Dunn's multiple comparisons test. *p* < 0.05 was considered significant.

## 3 Results

### 3.1 XR Irradiation‐Induced Jaw Malformation Is Ameliorated by Pretreatment With FA

**FIGURE 1:**
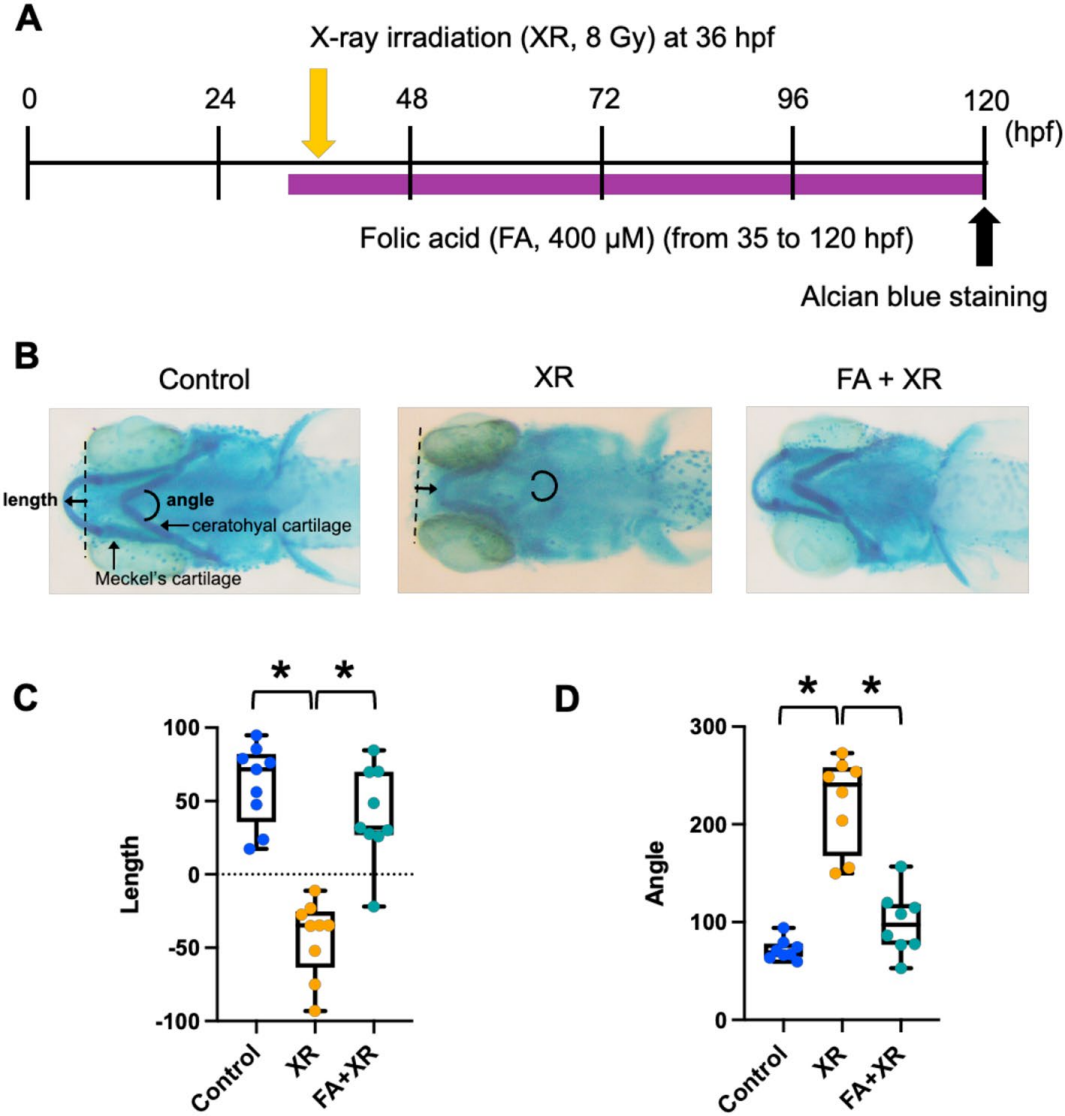
FA alleviates XR irradiation‐induced jaw malformation in zebrafish. (A) Schematic overview of the experimental design. (B) Representative images of Alcian blue‐stained zebrafish in the control, X‐ray irradiated (XR), and folic acid‐treated (FA) + XR (FA + XR) groups at 120 hpf. The dashed and curved lines indicate the length of Meckel's cartilage and angle of ceratohyal cartilage, respectively. (C, D) Quantification of the length of the vertical line from the tip of Meckel's cartilage to the connecting line between the tip of both eyes (C) and the ceratohyal cartilage angle (D). Box plots represent the median, interquartile range, minimum, and maximal values, and circles indicate individual fish. *N* = 9 (C) and 8 (D) zebrafish/group. *p < 0.05.

To assess the effect of XR irradiation and FA pretreatment on jaw development in zebrafish, three groups of embryos were left untreated (Control), XR irradiated at 36 hpf (XR), or treated with FA (400 μM) at 35 hpf and then XR irradiated at 36 hpf (FA + XR). Embryos were maintained until 120 hpf, stained with Alcian blue, and imaged (Figure 1A,B). Control embryos showed normally developed Meckel's cartilage and ceratohyal cartilage (Figure 1B, left panel), as evidenced by the location of the tip of Meckel's cartilage on the rostral side relative to the eye tips, and an interior angle of less than 180° at the tip of the ceratohyal cartilage. In contrast, embryos subjected to XR irradiation exhibited impaired protrusion of Meckel's cartilage beyond the eye tips and the interior angle of the ceratohyal cartilage was enlarged (Figure 1B, center panel). Notably, however, embryos pretreated with FA for 1 h before XR irradiation exhibited Meckel's cartilage protrusion and a ceratohyal cartilage angle similar to those seen in the untreated control embryo (Figure 1B, right panel). Quantification of both parameters confirmed the imaging observations and demonstrated a significantly shorter distance between the tip of Meckel's cartilage to the tip of the eyes (Figure 1C) and a significantly larger ceratohyal cartilage interior angle (Figure 1D) in the XR group compared with the control group. However, neither the length nor angle in the FA + XR embryos was significantly different from that in the control embryos (Figure 1C,D). We also examined the effects of 200 μM FA and found that this concentration tended to protect against XR irradiation‐induced jaw malformation (data not shown). These results indicate that XR irradiation at 36 hpf causes jaw malformation in zebrafish and that pretreatment with FA ameliorates the malformation.

### 3.2 XR Irradiation‐Induced ROS Formation Is Suppressed by Pretreatment With FA

**FIGURE 2:**
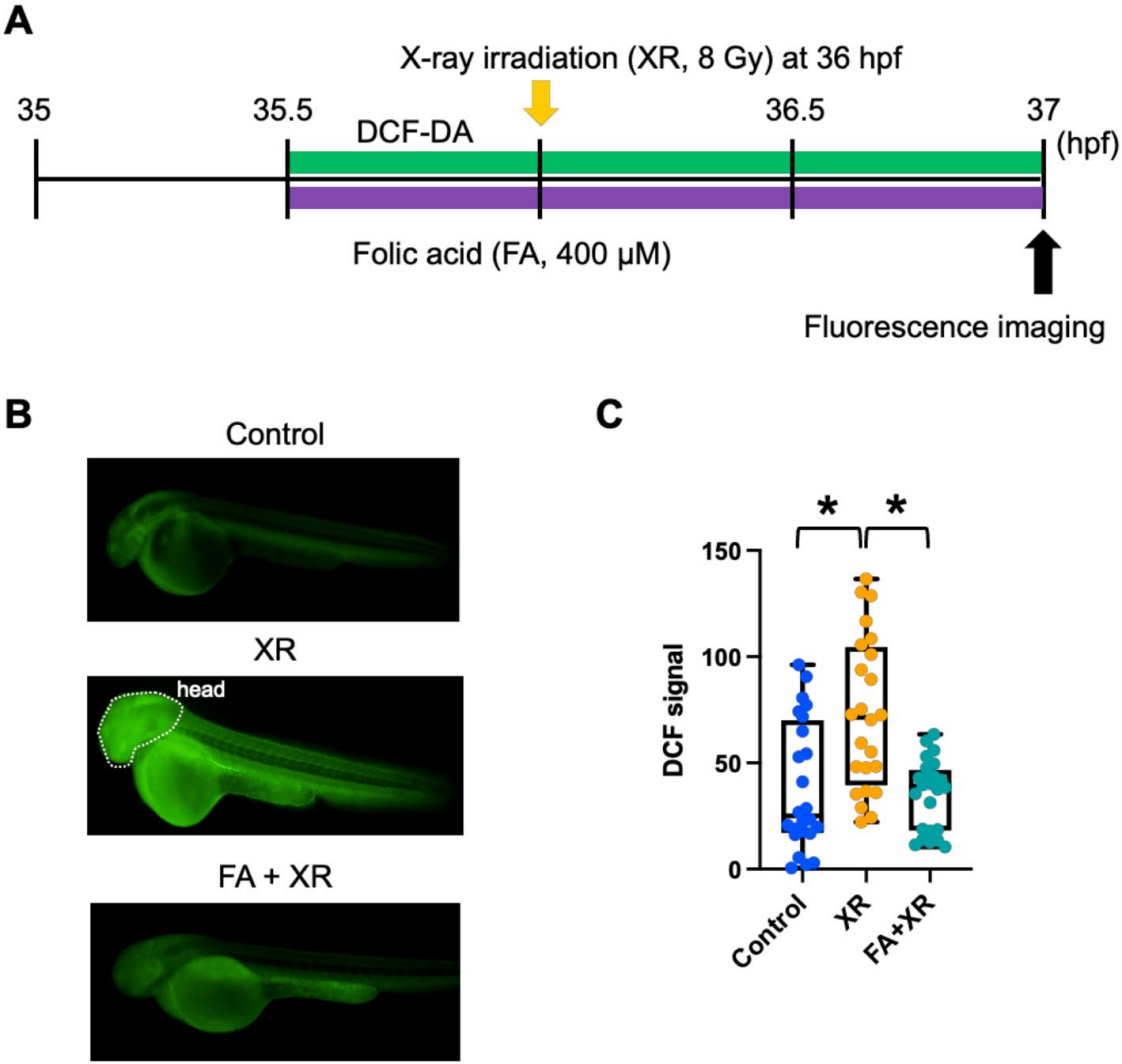
FA suppresses XR irradiation‐induced ROS formation in zebrafish. (A) Schematic overview of the experimental design. (B) Representative images of DCF‐DA fluorescence staining of zebrafish in the control, X‐ray irradiated (XR), and folic acid‐treated (FA) + XR (FA + XR) groups at 37 hpf. (C) Quantification of fluorescence intensity in the zebrafish head. Box plots represent the median, interquartile range, minimum, and maximal values, and circles indicate individual fish. *N* = 24 zebrafish/group. *p < 0.05.

Because oxidative damage is involved in ionizing radiation‐induced defects in embryonic development and FA is a scavenger of ROS, we investigated the effects of XR irradiation with or without FA pretreatment on ROS production in zebrafish embryos. At 35.5 hpf, FA (400 μM) was added to the FA + XR group of embryos and DCF‐DA was added to all three groups (control, XR, and FA + XR). DCF‐DA, a redox‐sensitive dye that is taken up by the cell and converted to a highly fluorescent form after interaction with ROS, has previously been used to detect ROS in zebrafish [25]. At 36 hpf, the XR and FA + XR embryos were subjected to XR irradiation, and all three groups were analyzed by fluorescence microscopy at 37 hpf (Figure 2A). Representative images of embryos from the treated groups (Figure 2B) demonstrate a marked increase in fluorescence signal in zebrafish exposed to XR compared with the control embryos (Figure 2B, top and middle panels), but the increase was not observed in embryos pretreated with FA before irradiation (Figure 2B, bottom panel). These microscopic observations were confirmed by quantification of the fluorescence signal in the zebrafish head, and demonstrated a significant increase in the fluorescence signal upon XR and a significant reduction back to control levels in the FA + XR group (Figure 2C). We also examined the effects of 200 μM FA and found that this concentration tended to suppress XR irradiation‐induced ROS production (data not shown). Taken together, these results suggest that XR irradiation increases ROS production in the zebrafish head, which is counteracted by FA pretreatment.

### 3.3 Transcriptome Analysis Identifies Genes Dysregulated by XR Irradiation and Normalized by FA Pretreatment

**FIGURE 3:**
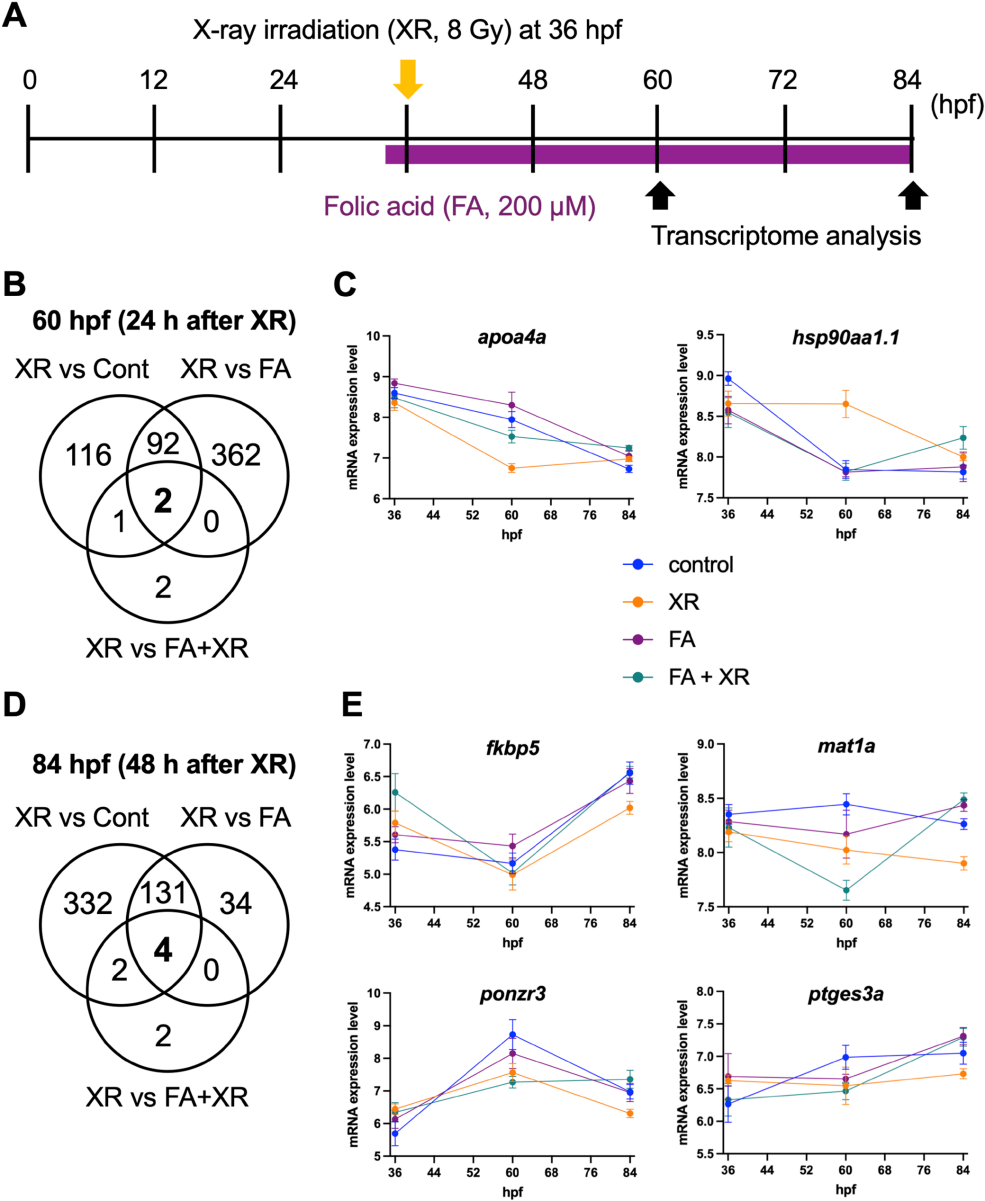
FA ameliorated dysregulated gene expression at 24 and 48 h after XR. (A) Schematic overview of the experimental design. (B) Venn diagrams showing the number of unique and shared differentially expressed genes (DEGs) in zebrafish at 24 h post‐XR irradiation, comparing XR‐irradiated (XR) versus control (Cont), XR vs. folic acid‐treated (FA), and XR versus FA + XR groups. (C) Expression levels of genes dysregulated by XR irradiation and ameliorated by FA at 24 h. (D) Venn diagrams showing unique and shared DEGs at 48 h post‐XR irradiation among the same three comparison groups. (E) Expression levels of genes dysregulated by XR irradiation and ameliorated by FA at 48 h. Data are presented as the means ± standard error. *N* = 6 zebrafish/group.

To further probe the molecular mechanisms underlying XR‐induced jaw malformation and its protection by FA, we performed transcriptome analysis of control, XR, FA, and FA + XR embryos. For the transcriptome analysis, we used 200 μM, but not 400 μM, FA to detect genes likely to be primary targets of FA. FA treatment was initiated at 35 hpf and XR irradiation was performed at 36 hpf, and embryos from all four groups were analyzed at 37 hpf (1 h post‐irradiation; Figure S1) or at 60 and 84 hpf (24 and 48 h post‐irradiation; Figure 3).

Transcriptome analysis at 37 hpf was performed to detect the early effects of XR and/or FA treatment on gene expression (Figure S1A). This analysis identified 25 and 77 genes that were differentially expressed in the XR vs. Cont groups and the XR vs. FA groups, respectively. However, no differentially expressed genes were identified in the XR vs. FA + XR groups, suggesting that the pretreatment with FA might be insufficient to influence gene expression within 1 h of XR irradiation (Figure S1B). There were no differentially expressed genes between the FA and Cont groups. Of the 25 and 77 genes differentially expressed in the control vs. XR groups and the control versus FA + XR groups, 17 genes were represented in both comparisons (Figure S1B). Gene ontology analysis of these 17 genes identified the enrichment of genes targeted by tumor protein p53 (Figure S1C), including v‐fos FBJ murine osteosarcoma viral oncogene homolog Ab (*fosab*), growth arrest and DNA‐damage‐inducible, alpha, a (*gadd45aa*), interferon stimulated exonuclease gene 20 (*isg20*), MDM2 proto‐oncogene (*mdm2*), pleckstrin homology‐like domain, family A, member 3 (*phlda3*), phosphoinositide‐3‐kinase, regulatory subunit 3a (*pik3r3a*), and ribosomal protein S27 (*rps27*). Figure S1D shows the expression of these genes in the four embryo groups and confirms the lack of effect of FA pretreatment before XR irradiation. These results indicate that the expression of tumor protein p53 target genes was increased within 1 h of XR irradiation but this was unaffected by pretreatment with FA.

Next, we examined gene expression at later times after XR irradiation by performing transcriptome analysis at 60 hpf (24 h post‐irradiation) and 84 hpf (48 h post‐irradiation; Figure 3A). At 60 and 84 hpf, 211 and 469 genes were differentially expressed in the XR versus Cont groups, respectively; 456 and 169 genes were differentially expressed in the XR versus FA groups, respectively; and 5 and 8 genes were differentially expressed in the XR versus FA + XR groups, respectively (Figure 3B). Two genes, apolipoprotein A‐IV a (*apoa4a*) and heat shock protein 90 alpha class A member 1 tandem duplicate 1 (*hsp90aa1.1*), were common to all three comparisons (XR vs. Cont, XR vs. FA, and XR vs. FA + XR) at 60 hpf, and the expression of these genes in the four zebrafish groups is shown in Figure 3B,C. In addition, four genes, FKBP prolyl isomerase 5 (*fkbp5*), methionine adenosyltransferase 1A (*mat1a*), plac8 onzin related protein 3 (*ponzr3*), and prostaglandin E synthase 3a (*ptges3a*), were common to the three comparisons at 84 hpf, and the expression of these genes is shown in Figure 3D,E. These six genes were subjected to gene ontology analysis and found to be significantly enriched with genes related to protein folding, including *hsp90aa1.1*, *fkbp5*, and *ptges3a* (data not shown).

## 4 Discussion

In this study, we used zebrafish as a model organism to examine the effect of XR irradiation on craniofacial development and to investigate a potential protective role for FA. Zebrafish were exposed to XR irradiation at 36 hpf, a time selected because it is a critical period in neural crest migration and chondrogenesis [26, 27]. We found that XR irradiation caused a marked reduction in Meckel's cartilage length and increased the ceratohyal cartilage angle, both of which disrupted craniofacial development. These phenotypes likely result from impaired chondrocyte growth, matrix deposition, and/or spatial organization [28]. However, pretreatment with FA starting 0.5–1 h prior to XR irradiation partially or completely prevented the defects in both parameters. We also analyzed ROS production in zebrafish using a redox‐sensitive fluorescent dye, DCF‐DA, and found that embryos treated with FA before XR irradiation had lower fluorescence signals compared with untreated XR‐irradiated embryos, indicating a reduction in oxidative stress burden by FA. These findings align with prior studies with zebrafish and substantiate the protective effect of FA against chemical and environmental stressors during gestation. For example, FA was shown to attenuate oxidative damage and developmental toxicity caused by arsenic exposure [29] and to ameliorate bisphenol A‐induced neurotoxicity and oxidative stress [30]. Folic acid can suppress ROS through multiple mechanisms, including direct scavenging and inhibition of NADPH oxidase‐mediated ROS production via reduced homocysteine levels ([31], Joshi, Adhikari, Patro, Chattopadhyay & Mukherjee, 2001). These findings suggest that FA plays a crucial role in protecting embryos against oxidative stress and developmental defects.

We also performed transcriptome analysis to investigate additional molecular mechanisms underlying the protective effects of FA against XR‐induced jaw malformation. These analyzes demonstrated that XR irradiation increased the expression of genes targeted by tumor protein p53. This finding is in agreement with previous studies showing that XR irradiation can activate tumor protein p53 and transcription of its target genes [32, 33]. Tumor protein p53 plays multiple roles in regulating biological processes, and its activation can be beneficial or detrimental, depending on the context. For example, tumor protein p53 activation may be beneficial during exposure to low levels of oxidative stress, due to increase the expression of antioxidant genes such as glutathione peroxidases and superoxide dismutases. In contrast, tumor protein p53 activation may be detrimental under conditions of high oxidative stress because of the induction of prooxidant genes such as tumor protein p53‐inducible gene 3 and proline oxidase [34, 35]. Our finding that FA pretreatment suppressed ROS production suggests that XR irradiation‐induced tumor protein p53 activation has at least some beneficial effects during zebrafish development. Of note, FA pretreatment did not affect the XR‐induced changes in gene expression occurring at 1 h post‐irradiation. This may be because FA modulate the function of tumor protein p53 on redox homeostasis without affecting the transcriptional activity.

Our transcriptome analyses performed at 24 and 48 h post‐irradiation identified dysregulated expression of a number of genes related to protein folding, including *hsp90aa1.1*, *fkbp5*, and *ptges3a*, and also showed that pretreatment with FA reduced the effects of irradiation on expression of several genes. These findings are also consistent with earlier studies demonstrating disruption of protein folding by XR irradiation [36] and its restoration by FA treatment [37]. Previous work suggests that oxidative stress can lead to endoplasmic reticulum stress and exacerbate protein misfolding [38].

FA also normalized the decreased expression of *apoa4a* and *mat1a* at 24 and 48 h after XR irradiation, respectively. *Apoa4* plays a major role in lipid transport and also has antioxidant and anti‐inflammatory properties [39]. In a mouse model of carbon tetrachloride‐induced acute liver injury, *Apoa4* overexpression mitigated oxidative damage and inflammation, whereas *Apoa4* deficiency exacerbated tissue injury [40]. Similarly, *Apoa4* was shown to attenuate oxidant‐induced apoptosis of PC12 cells, a rat pheochromocytoma cell line, by modulating intracellular glutathione redox balance [41]. FA supplementation in apolipoprotein E‐deficient mice delayed atherosclerotic lesion development, possibly by increasing *Apoa4* expression [42]. *Mat1a* encodes an enzyme responsible for the transfer of adenosyl moieties during the formation of S‐adenosylmethionine, which plays critical roles in many cellular processes, including DNA and protein synthesis. Impairment of *Mat1a* function results in decreased S‐adenosylmethionine production, leading to reduced glutathione synthesis and subsequent mitochondrial dysfunction and oxidative stress [43, 44]. Impairment of *Mat1a* also causes dysregulation of various biological methylation processes, including those of DNA and histones, which are essential for regulating gene expression [45].

Taken together, these results suggest that XR irradiation increases oxidative stress and dysregulates gene expression related to protein folding and biological methylation, and that pretreatment with FA ameliorates these effects. These results extend previous observations in mammalian models and provide a mechanistic foundation for exploring FA as a safe and cost‐effective radioprotective agent with potential implications for prenatal protection against oxidative stress in higher vertebrates, including humans.

## Funding

This work was supported by Japan Society for the Promotion of Science KAKENHI (23K06355), the Long‐range Research Initiative of the Japan Chemical Industrial Association (25‐1‐12), Ikuura Mariko Foundation, Mie University Graduate School of Medicine, the Japanese Cleft Palate Foundation.

## Conflicts of Interest

The authors declare no conflicts of interest.

## Supporting information


**Figure S1:** Tumor protein p53 target genes were increased at 1 h after XR with or without FA. (A) Schematic overview of the experimental design. (B) Venn diagram showing the number of unique and shared differentially expressed genes (DEGs) in zebrafish at 1 h post‐XR irradiation, comparing XR‐irradiated (XR) vs. control (Cont), XR vs. folic acid‐treated (FA), and XR vs. FA + XR groups. (C) Gene ontology analysis of 17 genes dysregulated by XR, revealing the enrichment of tumor protein p53 target genes. (D) Expression levels of seven tumor protein p53 target genes in the transcriptome analysis performed at 1 h post‐irradiation. Box plots represent the median, interquartile range, minimum, and maximal values, and circles indicate individual fish. *N* = 6 zebrafish/group.

## Data Availability

The data that support the findings of this study are available from the corresponding author upon reasonable request.
