# Supplementary material for: Folic Acid Alleviates X‐Ray Irradiation‐Induced Jaw Malformation in Zebrafish
Source: Congenit Anom (Kyoto). 2026 Jul 1;66(1):e70069. doi: 10.1002/cga.70069 (PMC13322707; doi:10.1002/cga.70069)

A

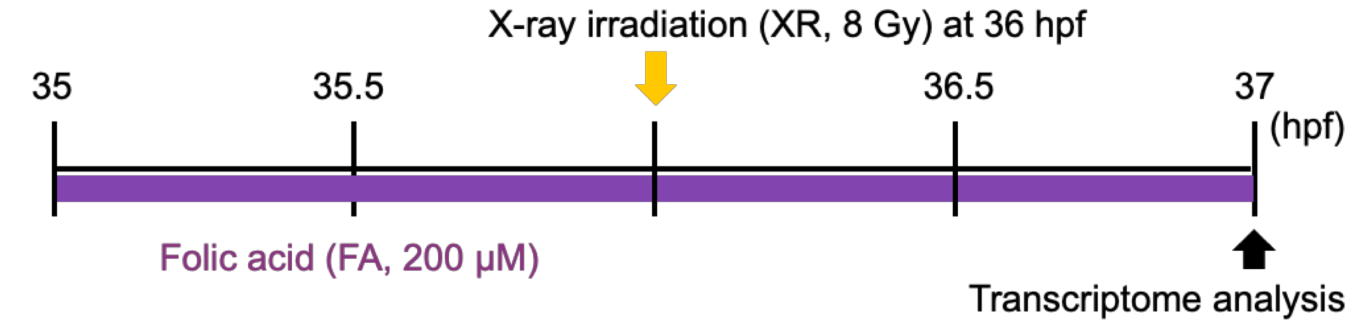

B

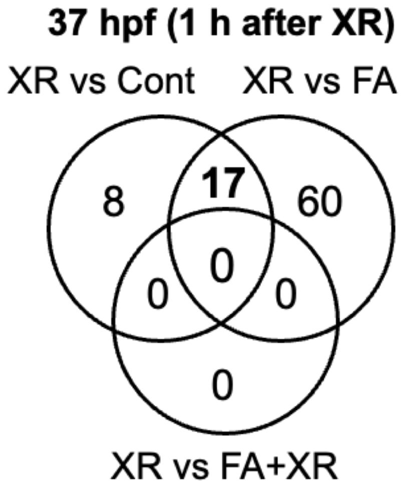

C

term_name	adjusted p value	intersections
p53; motif: RGRCWWGYCYNGRCWWGYYY	6.48E-04	*fosab, gadd45aa, isg20, mdm2, phlda3, pik3r3a, rps27l*
P53; motif: RGRCATGYCYRGRCATGYYY	5.92E-03	*fosab, gadd45aa, isg20, mdm2, phlda3, pik3r3a, rps27l*

D

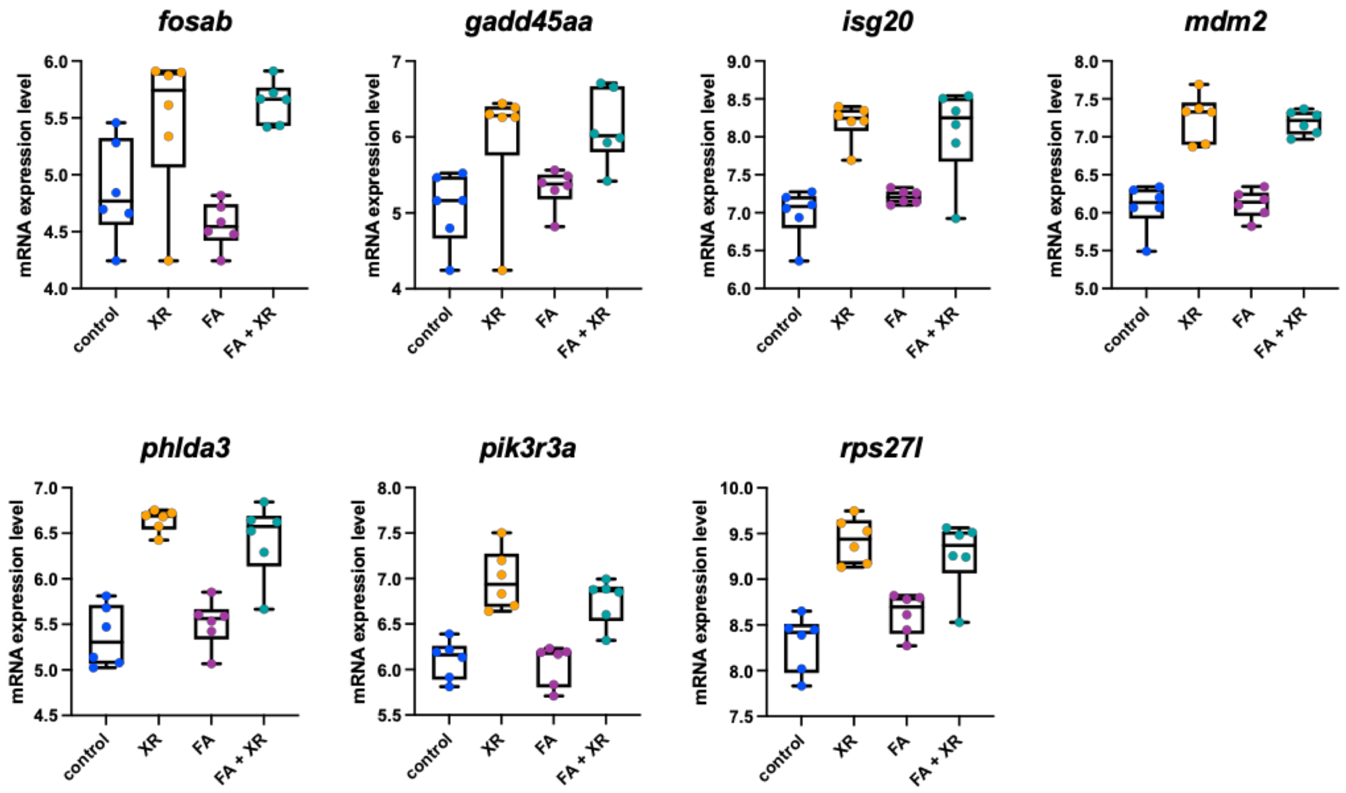

Supplement: Supplementary file 1 — Figure S1: Tumor protein p53 target genes were increased at 1 h after XR with or without FA. (A) Schematic overview of the experimental design. (B) Venn diagram showing the number of unique and shared differentially expressed genes (DEGs) in zebrafish at 1 h post‐XR irradiation, comparing XR‐irradiated (XR) vs. control (Cont), XR vs. folic acid‐treated (FA), and XR vs. FA + XR groups. (C) Gene ontology analysis of 17 genes dysregulated by XR, revealing the enrichment of tumor protein p53 target genes. (D) Expression levels of seven tumor protein p53 target genes in the transcriptome analysis performed at 1 h post‐irradiation. Box plots represent the median, interquartile range, minimum, and maximal values, and circles indicate individual fish. N = 6 zebrafish/group. [file CGA-66-0-s001.pdf]
